# Supplementary material for: Patterns of sex behaviors and factors associated with condomless anal intercourse during the COVID-19 pandemic among men who have sex with men in Hong Kong: A cross-sectional study
Source: PLoS One. 2024 Apr 4;19(4):e0300988. doi: 10.1371/journal.pone.0300988 (PMC10994335; doi:10.1371/journal.pone.0300988)
Supplement: Attachment — Submitted filename: Response to Reviewers_240304.docx [file pone.0300988.s002.docx]

**Comments from the editor:**

Please review your reference list to ensure that it is complete and correct. If you have cited papers that have been retracted, please include the rationale for doing so in the manuscript text, or remove these references and replace them with relevant current references. Any changes to the reference list should be mentioned in the rebuttal letter that accompanies your revised manuscript. If you need to cite a retracted article, indicate the article’s retracted status in the References list and also include a citation and full reference for the retraction notice.

**Response:** Thank you for your comment. We have reviewed the reference list. We slightly changed the information regarding reference No.1, No.5, and No.19 in the bibliography to make information provided correct. In addition, we deleted the reference No.9.

Thanks to the authors for submitting the revised version which much improvement. Please address a minor comment raised by the reviewer about the inconsistency of the numbers used in text and table in the results section.

**Response:** Thank you for your comment. We have revised the manuscript according to the comment raised by the reviewer.

**Comments from the reviewer:**

Title

- It is clear now that the study is a cross-sectional study.

**Response:** Thank you for your comment in the previous round.

Abstract

- The new version provides a better understanding of the terminologies and the research methods.

**Response:** Thank you for your comment in the previous round.

Methods

- The new version has more detailed information on the procedure.

**Response:** Thank you for your comment in the previous round.

- Sample size calculation: need a reference/references

**Response:** Thank you for your comment. We have revised the manuscript to provide references for information regarding sample size calculation.

Results

- Need to be consistent with numbers after coma. i.e “Table 2. Participants reported an average of 1.2 regular partners (SD = 1.34), 2.09 non-regular 288 partners (SD = 4.58), and 0.08 casual partners (SD = 0.80) during the COVID-19 period”. In the table, they used 1.24; in the description, it is 1.2. Meanwhile, for numbers, they used two numbers after the coma.

**Response:** Thank you for your comment. We have revised the manuscript to make sure that numbers of the present study are rounded to 2 decimal places and percentages of the present study are rounded to 1 decimal place.

We would like to take this opportunity to thank the reviewer and the editor who have provided very constructive comments on our paper. Your comments have helped us strengthen our arguments and enhance the quality of our manuscript. We greatly appreciate your review of our resubmission and look forward to your reply in due course. Should you have any enquiries, feel free to contact us. Thank you very much for your consideration.
